# The Importance of Interferon-Tau in the Diagnosis of Pregnancy

**DOI:** 10.1155/2021/9915814

**Published:** 2021-09-02

**Authors:** Alicja Kowalczyk, Ewa Czerniawska-Piątkowska, Marcjanna Wrzecińska

**Affiliations:** ^1^Department of Environmental Hygiene and Animal Welfare, Wrocław University of Environmental and Life Sciences, Chełmońskiego 38C, Wrocław, Poland; ^2^Department of Ruminant Science, West Pomeranian University of Technology, Ul. Klemensa Janickiego 29, 71-270 Szczecin, Poland

## Abstract

Several decades of improving dairy cattle towards unilateral utilization of dairy cattle led to enormous progress in the field of milk yield; however, it resulted in a number of unfavorable features, such as reproductive disorders, increased calf mortality, and reduced health. Most cases of embryo loss and/or lost pregnancies occur during the first four to five weeks of gestation; accurate detection for pregnancy during this period is likely to contribute to an improvement in gestation rates. A specific protein, interferon-tau (IFNT), stimulates interferon-stimulated genes (ISGs), and their expression increases during gestation within 21 days after insemination. In bovines, the early conceptus undergoes a phase of rapid growth and elongation before implantation, the latter occurring 2–3 weeks after fertilization. IFNT acts mainly in the endometrium of the luminal epithelium. It is a new type I interferon that regulates several genes encoding uterine-derived factors. They are crucial in the processes of preparing the uterus for placenta attachment, modifying the uterine immune system, and regulating early fetal development. Because IFNT is expressed and induces ISGs in the endometrium during pregnancy recognition, it was reasoned that surrogate markers for pregnancy or IFNT might be present in the blood and provide an indicator of pregnancy status in cattle.

## 1. Introduction

Enormous success in net milk production has been achieved due to the selection of dairy cattle. This selection was aimed at increasing the milk yield of cows [1]. However, cows characterized by high milk yield show reduced fertility. There is a negative correlation between productive and reproductive rates [2, 3]. This is noticeable in high-yield cows that have a lower calving rate (~34%). In these cows, most miscarriages occur within 16 days of insemination [4]. However, early pregnancy loss is not detectable. It can be detected 21 days after insemination when the cow shows signs of estrus. If pregnancy is not detected at an early stage, it results in an extension of the calving interval and consequently reduced milk production. It causes economic losses [5, 6]. Improving reproductive performance by increasing the pregnancy rate and shortening the calving intervals is a consequence of early pregnancy detection in cows after insemination [7].

In the first four to five weeks after fertilization, most cases of embryo loss and/or pregnancy loss are observed. The precise detection of gestation at this time can improve the pregnancy rate [6]. So far, various studies have been conducted for the development of a rapid and straightforward method for the detection of bovine pregnancy [6, 8–13], but most of them, such as rectal palpation, ultrasonography, and monitoring of circulating progesterone, utilize molecular tests to indicate conception success; however, pregnancy is then confirmed by the detection of the fetus approximately 4 to 5 weeks after insemination [14–16].

### 1.1. Interferon-Tau

A specific protein such as interferon-tau (IFNT) stimulates interferon-stimulated genes (ISGs), and their expression increases during gestation within 21 days after insemination [17–19]. Cows that did not become pregnant after the insemination were identified by the absence of suboptimal detection of embryo-induced ISGs in the maternal circulation on days 18-20. In order to reduce the calving interval and improve the profitability of the dairy activity, we can use quick resynchronization of the oestrus cycle of cows that did not become pregnant [20].

IFNT signals use classical Type I interferon receptors 1 and 2 (IFNAR1 and IFNAR2) when activating the JAK-STAT signaling pathway [21]. IFNAR1 and IFNAR2 are expressed in the ovine uterus on days 14-15 in nonpregnant and pregnant ewes [22]. IFNT can also signal via mitogen-activated protein kinase (MAPK) and phosphatidyl inositol 3-kinase (PI3K) [23]. IFNT acts to rescue the *corpus luteum* (CL) and maintain the pregnancy. However, IFNT is not stimulated by viral infection [24], and transcriptional factors that have control of IFNA and IFNB do not regulate IFNT expression [25]. The expression of IFNT gene is controlled by transcription factor ETS2 [26]. Additionally, IFNT expression is also increased in the elongation phase by combinatory trophoblastic gene regulators DLX3, CDX2, and GATA2/3 [27, 28].

### 1.2. Maternal Recognition of Pregnancy

Maternal recognition of pregnancy can be defined as the physiological process whereby the conceptus signals its presence to the maternal system and prolongs the lifespan of the ovarian CL [29]. This process in cows requires that the conceptus be elongated to ensure the correct amount of IFNT. Then, pregnancy is diagnosed, and the luteolytic mechanism of the endometrium is inhibited [24, 30–32]. The effect of IFNT on the endometrium is based on the antiluteolytic activity which maintains CL function and progesterone secretion. It is crucial for the growth and development of the fetus during pregnancy. Mononuclear trophectoderm cells of ruminant embryos (embryonic and extraembryonic membranes) secrete interferon tau. This type of interferon is characterized by properties such as antiviral, antiproliferative, and immunomodulatory.

In bovines after conception, there is a phase of rapid embryo growth and elongation. Then, 2-3 weeks after fertilization occurs the implantation [33, 34]. Around the 13th day of pregnancy, the embryo lengthens. Then, the embryo changes from spherical to ovoid, then tubular and filamentous [35, 36]. In order to ensure the secretion of suitable concentrations of IFNT, as well as the recognition by the mother of pregnancy, it is necessary to elongate the embryo. The elongation is also aimed at increasing the maximum area of vascular exchange with the mother's tissues after the implantation [37, 38]. The loss of the embryo may be due to the inability to elongate. It may also affect the reproductive failure of cows [38–40].

Maternal recognition of the pregnancy period where bovine conceptus secretes IFNT to signal its presence within the uterus occurs between days 12 and 26 of pregnancy [41]. However, recent researches have shown that the bovine conceptus signals to the mother previously to this period. Bovine oviduct epithelial cells (BOECs) stimulate in vitro bovine embryos on day 4 to produce IFNT, which then acts on immune cells to promote an anti-inflammatory response in the oviduct. However, in this period, the IFNT was not able to stimulate ISG expression in BOECs [42]. Studies report that the endometrial preparation to embryonic receptivity can occur as early as day 4 due to an increase in steroid hormone concentration from large follicles [43]. Moreover, on day 7 after artificial insemination, embryo-dependent factors are already able to modulate ISGs, prostaglandin biosynthesis, and water channel and solute transport pathways in the endometrium at the uterotubal junction of the uterine horn ipsilateral to the CL [44], demonstrating that IFNT secretion may occur before the classic maternal recognition period of pregnancy.

**Figure 1:**
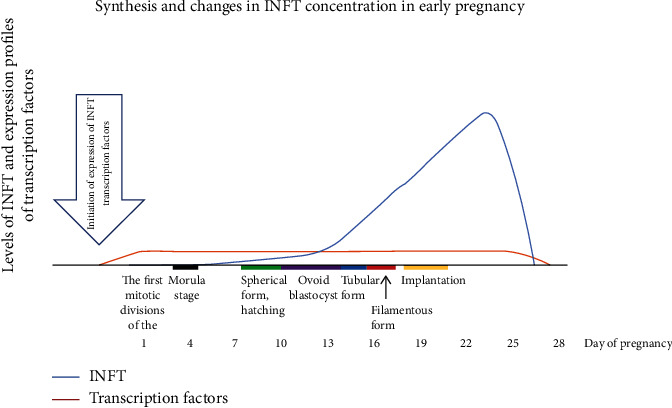
Diagram of the synthesis and changes of INFT levels during early pregnancy in a cattle after the expression of INFT transcription factor profiles (source: [45, 46]).

Figure 1 (Figure 1) shows a diagram of the synthesis and changes in INFT levels during early pregnancy in cattle.

### 1.3. Interferon-Stimulated Genes

There are three main IFN subclasses, namely, types I, II, and III. Type I IFNs contain at least 9 subfamilies, including IFN-alpha (IFNA), beta (IFNB), delta (IFND), omega (IFNW), epsilon (IFNE), kappa (IFNK), tau (IFNT), zeta (IFNZ), and X (IFNX) [47, 48]. The type I family probably has arisen from the type III family due to the fact that mammalian lines diverged from birds and reptiles about 300 million years ago [49, 50]. In the bovine genome, seven of these nine IFN subfamilies (IFNA, IFNB, IFNE, IFNK, IFNW, IFNT, and IFNX) have been identified [48].

IFNT acts mainly in the endometrium of the luminal epithelium. It is a new type I interferon. IFNT regulates several genes encoding uterine-derived factors. They are crucial in the processes of preparing the uterus for placenta attachment, modifying the uterine immune system, and regulating early fetal development [51–53].

The unique trophoblast-specific expression in ruminants is an essential feature that helps to distinguish IFNT from another type I IFNs. From the start of transcription of the IFNT gene, the first 400 bases and upstream are exceptional for the IFNT gene. There are no virus-induced transcriptional elements in this region [54]. However, in the 5′ promoter/enhancer region, there are several elements that control trophoblast transcription, including ETS2 and DLX3 [35, 55]. The transcription of IFNT into trophoblast cells may be restricted by the 5′ UTR. These cells are the outermost cells of the developing fetus that will form the outermost layer of the placenta. At the morula and blastocyst stage (6-7 days of gestation of the cow), expression in the bovine embryos begins. Expression continues until trophoblast cells adhere to the uterine epithelium (~day 16 in cattle) [56, 57]. In the morula as well as the blastocyst stage, the production of IFNT begins. However, it increases with the elongation of the embryo (days 14–17 in cattle).  Table 1 shows the changes in INFT content in cattle in the early stages of pregnancy.

All IFNT paracrine actions block luteolytic PGF pulses, and they also induce expression of ISGs that may have a biological function during maternal recognition and establishment of pregnancy [58].

### 1.4. The Role of IFNT and ISGs in Pregnancy Diagnosis

Surrogate markers of pregnancy or IFNT can be used as an indicator of the status of pregnancy in cattle because they may be present in the blood. IFNT may be a good marker as it is expressed and induces ISG in the endometrium during recognition of gestation.

The IFNT analyses did not reveal a sensitivity exceeding the low ng levels from studies based on ISGs. Detecting ISGs as indicators of pregnancy in the blood is not a new practice [59]. Moreover, there are no ISG-based protein biomarkers available to determine pregnancy status from blood. In pregnant sheep 15 to 30 days after insemination, higher levels of MX1 mRNA in PBMC were found compared to nonpregnant sheep [60]. Also in cattle, a similar relationship was observed between pregnancy and ISG in PBMC [61, 62]. In pregnant cows, mean blood levels of ISG15 mRNA are higher from days 15 to 32 od gestation (maximum level is on the 20th day of gestation) compared to fertilized nonpregnant cows. The identification of ISG15 mRNA in PBMC for several days gave a more accurate prediction of gestation than with a single assay. On one hand, studies in cattle showed an increase in ISG in heifer leukocytes in response to pregnancy on day 18. On the other hand, it was not observed in lactating dairy cows [63]. A beef cow study showed an increase in ISG mRNA between days 15 and 22 od gestation. The maximum concentration was reached on the 20th day of pregnancy [64]. This showed that ISG leukocyte mRNA levels along with CL ultrasound on day 20 of pregnancy are a more precise tool for predicting pregnancy.

In pregnant Holstein-Friesian cows, researchers defined the shortest time to detection of pregnancy. They defined it by measuring ISGs in blood on days 17 and 18 after AI in nulliparous, primiparous, and multiparous cows as well [63]. Primiparous cows had the highest number of ISG on the 20th day. This correlates with maximal production of IFNT by the elongated embryo. ISG-based detection may be more feasible with nulliparous cows as high false-positive rates were obtained during the lactation period. Higher levels of PBMC ISG15 and RTP4 were shown in Holstein primiparous cows than in multiparous cows on day 19 [65]. The differences in ISG expression between heifers or primiparous and multiparous cows may be explained by the length of the embryos. Because, the length of the embryos is longer in heifers than in cows during pregnancy diagnosis, this influences the amount of IFNT produced [66]. Also, another possibility for better peripheral expression of ISG in heifers may be that they are smaller than multiparous cows. This may have an impact on systemic IFNT levels and consequently on the ISG response in leukocytes. An attempt to improve the accuracy of early pregnancy diagnosis has been made by Green et al. They used the ratio of ISG expression on day 18 post AI to ISG expression based on a control blood sample collected before AI [63].

## 2. Conclusion

Interferon-tau (IFNT) is the main signal for the maternal recognition of pregnancy in ruminants and exerts its effects by stimulating the expression of interferon-stimulated genes. The process of embryo lengthening significantly affects the production of an appropriate amount of IFNT, and its detection in peripheral fluids located distal from the uterine vein may soon become a reliable and competitive candidate for a biomarker for early pregnancy detection.

## Figures and Tables

**Table 1:** Changes in INFT content in cattle until embryo implantation.

Stage of pregnancy	INFT content	References
Initial blastomere cleavage (1-6 days)	Forms of trophectoderm	[38, 46]
Blastocyst formation (7-8 days)	Forms of trophectoderm	
Embryo spherical form (9-12 days)	Increase in INFT content, INFT expression	
Extending the embryo of the filamentous form (15-18 days)	Big increase in INFT content	
Implantation (21-24 days)	The highest levels of INFT achieved, maternal diagnosis of pregnancy	
>24 days	A sharp decrease in the INFT content, an exponential increase in the trophectoderm layer of a bovine embryo	

## Data Availability

Data availability is not applicable.
